# Effectiveness of electroacupuncture for thin endometrium in infertile women: study protocol for a single-blind, randomized controlled trial

**DOI:** 10.1186/s13063-021-05029-7

**Published:** 2021-01-21

**Authors:** Fangyuan Li, Hua Lu, Xinxin Wang, Qi Zhang, Qianchen Liu, Tong Wang

**Affiliations:** 1 College of Clinical Medicine, Chengdu University of Traditional Chinese Medicine, NO.37 Shi-er-qiao Road, Chengdu, 610075 Sichuan Province People’s Republic of China; 2 Hospital of Chengdu University of Traditional Chinese Medicine, NO.39 Shi-er-qiao Road, Chengdu, 610075 Sichuan Province People’s Republic of China; 3 College of Acupuncture and Tuina, Chengdu University of Traditional Chinese Medicine, NO.37 Shi-er-qiao Road, Chengdu, 610075 Sichuan Province People’s Republic of China

**Keywords:** Electroacupuncture, Thin endometrium, Effectiveness, RCT

## Abstract

**Background:**

Thin endometrium negatively impacts the reproductive function. Current treatments for thin endometrium do not always improve endometrial receptivity. Preliminary evidence suggests that electroacupuncture could have potential therapy for thin endometrium in infertile women. Thus, this randomized controlled trial was designed to test whether electroacupuncture can improve endometrial receptivity in infertile women with thin endometrium.

**Methods:**

This study is a randomized, single-blinded, controlled, clinical trial. A total of 142 eligible patients will be recruited and randomly assigned to the electroacupuncture (EA) group or the sham electroacupuncture (SEA) group in a 1:1 ratio. Participants will receive 36 sessions over three menstrual cycles (12 weeks in total), with the same acupoint prescription. The primary outcome of this trial is endometrial thickness in the midluteal phase. The secondary outcomes include endometrial pattern, resistance index (RI) and pulsatility index (PI) of bilateral uterine artery and endometrium blood flow, serum estradiol (E2) and progesterone (P), and pregnancy rate. The pregnancy rate will be evaluated during a 6-month follow-up after completion of the trial. All other outcomes will be evaluated before treatment, during the treatment of 1st, 2nd, and 3rd menstrual cycle, and 6 months after treatment.

**Discussion:**

If the outcome confirms the effectiveness of electroacupuncture for thin endometrium in infertile women, this treatment will be proposed for application in clinical practice.

**Trial registration:**

Chinese Clinical Trials Registry ChiCTR2000029983. Registered on 18 February 2020

**Supplementary Information:**

The online version contains supplementary material available at 10.1186/s13063-021-05029-7.

## Background

Previous research has shown that endometrial thickness is related to endometrial receptivity [1]. An inadequate endometrium can be considered as a main hindrance to fertility [2]. It has been shown that thin endometrium leads to infertility [3]. Likewise, a “thin” endometrium is associated with implantation failure and low implantation rates [1, 4]. Globally, 8 to 12% of reproductive age couples suffer from infertility which has become a major global public health issue [5]. Despite the advances in assisted reproduction, many patients with thin endometrium repeated implantation failure [6]. It has been reported that nearly two-thirds of failed implantation in IVF (in vitro fertilization) are caused by poor endometrial receptivity [7]. Incidences of thin endometrium in ovarian stimulation cycles and natural cycles are 38–66% [8] and 5–25% [9], respectively. In fresh or frozen-thawed embryo transfer cycles, thin endometrium often leads to cancelation of ET and cryopreservation of all the embryos [10]. A thin endometrium is associated with low conception rate [11–13], but also to a higher probability of spontaneous abortion and ectopic pregnancy [14]. The probability of clinical pregnancy was 23.3% for an endometrium measuring ≤ 7 mm, which was significantly lower than that of endometrium measuring > 7 mm (48.1%) [13]. The lowest pregnancy rates were associated with endometrial thickness of < 7 mm in 768 consecutive medicated FER (frozen embryo replacement) cycles [15]. Hence, thin endometrium is often defined as less than 7 mm during the late follicular phase or after ovulation [16, 17]. Endometrial receptivity appears to be the bottleneck of the reproductive process [18]. Therefore, improving endometrial receptivity among infertile patients with thin endometrium is urgently needed in reproductive medicine.

The endometrium, a unique cyclic tissue regeneration system, depends on the cyclical growth and regression of blood vessels that supply the endometrium [19]. Previous works showed that angiogenesis which depends on the expression of angiogenic factors (VEGF, FGF, and angiopoietin families) is a critical factor in determining the endometrial receptivity during the implantation window (IW) [20]. Thin endometria are characterized by high uterine blood flow impedance, poor growth of glandular epithelium, decreased expression of vascular endothelial growth factor (VEGF), and poor vascular development [21]. Current therapeutic strategies for thin endometrium include long-term administration of exogenous estrogen, low-dose of aspirin, vaginal sildenafil citrate application, transvaginal endometrial perfusion of granulocyte colony-stimulating factor (G-CSF), autologous platelet-rich plasma infusion (PRP), stem cell therapy, vitamins C and E, and L-arginine supplement [8, 22]. Despite the vast diversity of treatment, most of these treatment options result in a minor change in the endometrium thickness and pregnancy rate [22]. Currently, there is no one acceptable approach to treat thin endometrium [23], thus the need for an alternative and complementary therapy for patients with thin endometrium.

Acupuncture may have a potential therapeutic effect on thin endometrium in infertile women. Pulsatility index (PI) in the uterine arteries is considered valuable in assessing endometrial receptivity, and it was found to have decreased after EA treatment [24]. EA has been found to improve endometrial angiogenesis during peri-implantation period by increasing the expression of VEGFR2/PI3K/AKT and VEGFR2/ERK signaling pathways in COH rats [25]. Estrogen promotes the reconstruction of the endometrial vascular system and the repair of damaged endometrium by binding to the estrogen receptor (ER) ligand [26]. A significant increase in endometrial thickness and the number of endometrial glands after EA treatment, probably due to up-regulation of serum E levels, and different regulation of the sex steroid receptors ERα and ERβ [27]. EA also plays an effective auxiliary role of supporting bone marrow mesenchymal stem cell (BMSCs) in repair of thin endometrium [28]. Furthermore, acupuncture may ameliorate the uterine environment by increasing the glandular area and expression of receptivity markers such as LIF and OPN proteins [29]. Clinical studies have found that acupuncture may improve the high-quality embryo rate [30] and clinical pregnancy rate [31] by improving endometrial blood flow state and the uterine environment. Although acupuncture has initially been shown to improve endometrial receptivity and pregnancy rates during the process of assisted reproduction, the associated evidence base is poor [32]. Thus, well-designed RCT should be conducted based on a rigorous methodology to evaluate the efficacy of EA in improving endometrial receptivity for thin endometrium in infertile women.

## Methods/design

### Study design

**Fig. 1:**
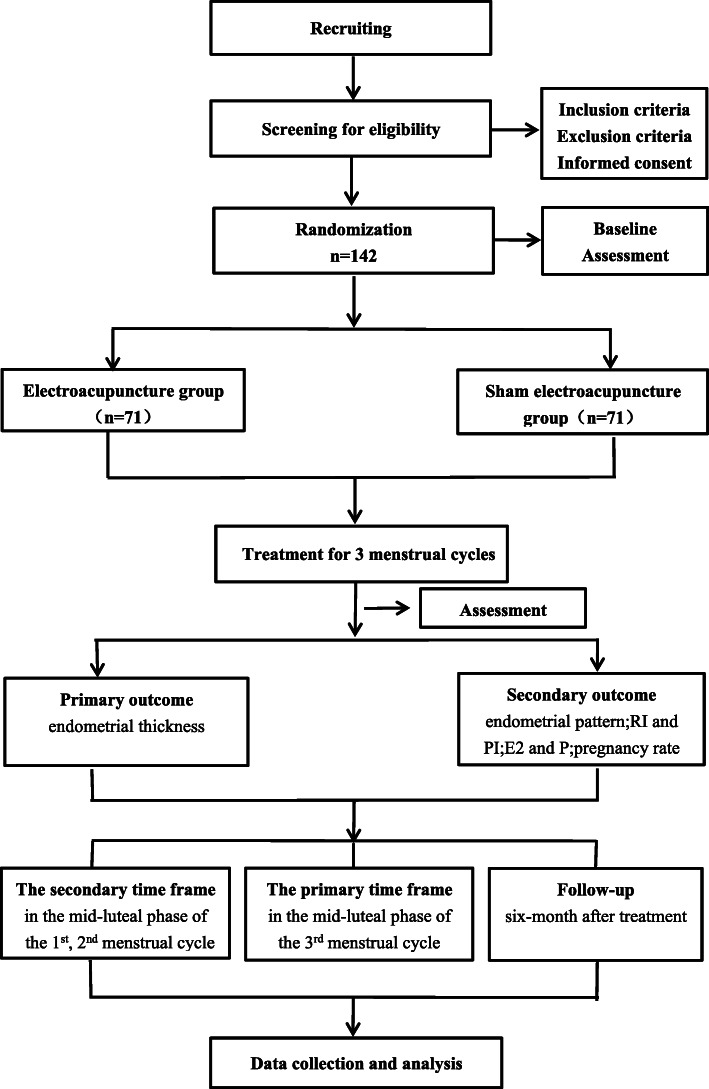
Trial flow chart

A single-center, patient-blinded, randomized controlled trial (RCT) was devised following the Consolidated Standards of Reporting Trials (CONSORT) statement [33], the Standardized Protocol Items: Recommendations for Interventional Trials (SPIRIT) guidelines [34], and the Revised Standards for Reporting Interventions in Clinical Trials of Acupuncture (STRICTA) [35]. If participants are eligible and agree to participate, they will be randomly assigned to electroacupuncture group (EA) or sham-electroacupuncture (SEA) group in a 1:1 ratio. The trial will be conducted in the Department of gynecology, Chengdu University of Traditional Chinese Medicine Affiliated Hospital. The flowchart is shown in Fig. 1.

### Ethics

The trial will be carried out in accordance with the Declaration of Helsinki [36]. Ethical approval has been obtained from the Sichuan Regional Ethics Review Committee on Traditional Chinese Medicine with an ethics approval number of 2019KL-072. The work reported in this article is registered with an identifier (ChiCTR2000029983) by the Chinese Clinical Trial Registry.

### Participants

The diagnostic criteria for thin endometrium are based on data provided in our previous studies: endometrial thickness (EMT) < 7 mm in the mid-luteal phase (7 days after the day of ovulation or on day 21 of an unstimulated ovarian cycle) [8, 15, 17]. Infertility will be diagnosed as the failure to conceive for at least 12 months of unprotected sexual intercourse [37].

### Inclusion criteria

Patients who conform to the following criteria will be included:

1. Females, aged between 20 and 45 years;
2. Meets the diagnosis of infertility and is trying to conceive;
3. EMT < 7 mm in the midluteal phase;
4. Willing to participate in this study and sign an informed consent form.

### Exclusion criteria

1. Infertility induced by an ovulation disorder, severe endometriosis, Asherman’s syndrome, luteal phase deficiency, fallopian tube obstruction, or male infertility;
2. Pregnancy or lactation;
3. History of psychiatric disorders (serious anxiety and depression, schizophrenia), coagulation disorders, blood infections (viral hepatitis, HIV), malignant tumor, diabetic neuropathy or complicated with serious heart, kidney or liver disease;
4. A cardiac pacemaker, metal allergy, or artificial joint;
5. History of acupuncture treatment in the last 3 months or use of estrogen, progestin in the past 4 weeks;
6. Participation in other clinical trials within the past 3 months.

### Dropout criteria

Patients who quit the study voluntarily or have poor clinical compliance during observation period or found to not conform to the inclusion criteria after enrolment will be considered as having dropped out.

### Recruitment strategies

Infertile female participants with thin endometrium will be recruited either through advertisements on notice boards in Chengdu University of Traditional Chinese Medicine Affiliated Hospital or advertisements in hospital social media (WeChat). Any interested patients will reach the researcher by telephone, email, or WeChat. If they meet the study criteria, they will be invited for a face-to-face interview for baseline screening with the researcher. The researcher will explain the study in detail, including potential benefits and risks. Once a participant agrees to participate in the study, informed consent will be obtained before randomization. Participants consent forms is shown in Additional file 1.

### Randomization

The participants will be randomly assigned to electroacupuncture group or sham-electroacupuncture group in a 1:1 ratio. Randomization will be performed based on a random list of numbers generated by SPSS21.0 software (International Business Machines Corp., Armonk, NY). Allocation will be conducted by a designated researcher who has no contact with any participant and is not involved in the data collection or analysis. Allocation will be concealed in sequentially numbered, opaque, and sealed envelopes containing the randomization assignments. The appropriate numbered, opaque, sealed envelope will be opened after each participant meets eligibility criteria and signs informed consent. The allocation concealment to the doctors is not feasible for trials on acupuncture. The acupuncturist will not be allowed to reveal any information about treatment procedures and outcomes to the participants or the assessor.

### Blinding

The specific nature of the intervention makes it impossible to blind the acupuncturist. However, the participants, data collection staff, and data analysts will be blinded. To maximize blinding of participants, placebo needles and sham electroacupuncture design will be used in the sham-electroacupuncture group. Adhesive pads will be applied to both EA and SEA groups to ensure that the participants are unaware of the differences between the two acupuncture treatments. Each participant will receive intervention in a personal space separated by bed curtain and no communication will be allowed between participants. During the intervention period, the acupuncturist and the data collection researcher will not be allowed to exchange information on grouping, treatment procedures, and outcomes. Blinded evaluation and blinded statistical analysis will be emphasized during the data collection and analysis stage.

### Interventions

#### Study flow

The treatments will be performed by one licensed acupuncturist who has at least 5 years of experience with acupuncture. All participants will be treated in supine position on the treatment couch. Treatment will be conducted during three menstrual cycles, at a frequency of 3 sessions per week (once every 2–3 days) in both intervention and control groups after randomization with each group receiving 30 min per session. If a woman becomes pregnant during the intervention phase, the intervention will be terminated. Six months after completion of the treatment, follow-up will be performed by telephone or WeChat to ask if the participants are pregnant.

#### Electroacupuncture (EA) group

To improve endometrial receptivity in infertile patients with thin endometrium, electroacupuncture prescription will be developed based on clinical practice and literature review. The obligatory acupoints will include bilateral Tianshu (ST25), Zigong (EX-CA1), Sanyinjiao (SP6), Guilai (ST29), individual Zhongji (RN3), and Guanyuan (RN4). Additionally, sets of adjunct acupoints will be alternately selected by the acupuncturists based on the periodic therapy of Traditional Chinese Medicine: bilateral Xuehai (SP10) and Diji (SP8) will be needled during menstruation; bilateral Taichong (LR3) and Taixi (KI3) will be needled after menstruation; bilateral Taichong (LR3), Hegu (LI4), and Zusanli (ST36) will be needled during the ovulatory period; individual Qihai (RN6) and bilateral Zusanli (ST36) will be needled before the next menstruation. Acupuncture points will be identified according to the method of point location issued by the World Health Organization. Stick adhesive pads (15 mm) will be placed at all points. After sterilization of the skin with alcohol wipes, single-use, stainless, sterile needles (gauge 0.25 × 40 mm or 0.25 × 60 mm; Hwato, Suzhou, China) will be needle inserted.

In a supine position, the needle will be inserted 40 mm vertically into the muscles of the abdominal wall through the pads for ST25, ST29, RN4, and RN3. EX-CA1 and RN6 will be inserted vertically to a depth of 25 mm in the abdomen. The needle will then be inserted vertically at SP6, SP10, and SP8 to a depth of 25 mm. LR3 will be punctured vertically to a depth of 15 mm into the skin, while LI4 and ST36 will be punctured perpendicularly to a respective depth of 25 mm and 40 mm, respectively. Deqi sensation (including numbness, soreness, distention, heaviness) will be achieved through lifting, thrusting, and rotating for 10 s on each point. An electric stimulator (HANS-200E acupoint nerve stimulator, Nanjing Jisheng Medical Co, Ltd) will be applied to bilateral ST25 and ST29 with disperse-dense waves of 2/100 Hz frequencies for 30 min. The current intensity will be increased until the needles start vibrating slightly. After a 30-min retention period, all needles, electrode clamps, and stick adhesive pads will be removed.

#### Sham-electroacupuncture (SEA) group

Participants in the sham electroacupuncture group will receive sham EA on therapeutic acupoints without skin penetration or electrical stimulation [38]. The procedures, frequency, and duration of treatment will be the same as that of the electroacupuncture group. Blunt needles which are similar to Streitberger needles device will be used [39]. This type of needle can provoke a needling sensation quite similar to real acupuncture as soon as it touches the skin, without insertion. The acupuncturists should pretend to manipulate the needles slightly with an even lifting, thrusting, and twisting method for 10 s on each point as well. No Deqi sensation will be induced. Similarly, the needles on bilateral ST25 and ST29 will be connected to a sham electric stimulator with the same parameters as the EA group, but the wire will be cut thus no current intensity. The light seen and the sound of the pulse generator heard will be the same as the EA group.

### Outcome measures

#### Primary outcome

The primary outcome will be endometrial thickness in the mid-luteal phase of the 3rd menstrual cycle (the primary time frame), the 1st menstrual cycle and 2nd menstrual cycle as well as 6 months after completion of the treatment (the secondary time frame) compared with baseline (in the − 1st menstrual cycle).

#### Secondary outcome measures

The secondary outcomes include the following four aspects: (1) endometrial pattern, (2) RI and PI of bilateral uterine artery and endometrium blood flow, (3) serum estradiol (E2) and progesterone (P), and (4) pregnancy rate. The first three of the secondary outcomes will be evaluated in the mid-luteal phase of 3rd menstrual cycle (the primary time frame) and the 1st menstrual cycle and 2nd menstrual cycle, as well as 6 months after treatment (the secondary time frame) in comparison with the baseline (in the − 1st menstrual cycle). The pregnancy rate will be assessed during a 6-month follow-up after completion of the trial.

A certified sonographer will measure the endometrial thickness and the first three secondary outcomes through transvaginal ultrasound. A laboratory physician will test the serum estradiol and progesterone through radioimmunoassay. They will be trained and assessed before the trial.

#### Assessment of adverse events

Risks associated with this study are minimal. However, some possible adverse events may include bleeding, hematoma, fainting, continuous post needling pain, local infection, and other symptoms. Participants will be asked to report any adverse events during the trial, and these will be recorded on an adverse event report form in the case report form (CRF). Once a serious adverse event occurs, the researcher will terminate the trial and rescue procedures will be initiated at once. A written report to the Ethics Committee should be submitted immediately. The overview of the outcome measurement at different time points is shown in Table 1.

**Table 1:**
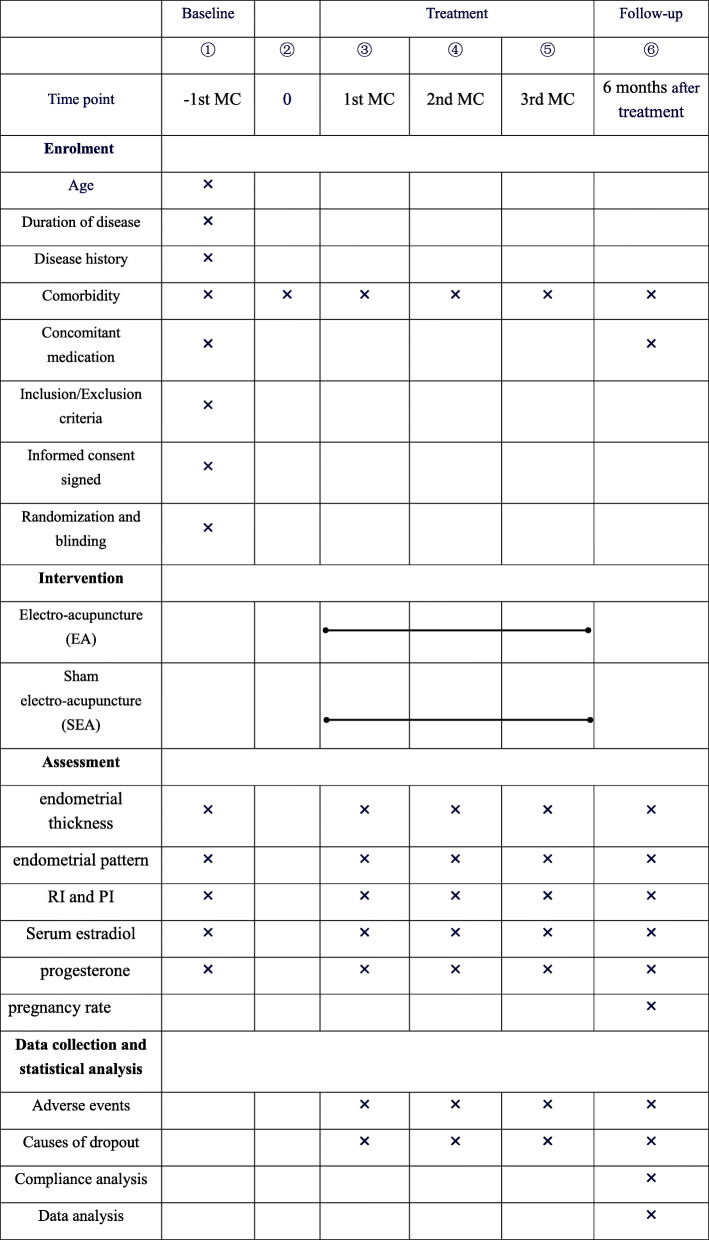
Data collection points

The schedule of enrolment, interventions, and assessments

*MC* menstrual cycle, *PI* pulsatility index, *RI* resistance index

#### Sample size

There is insufficient previous data on endometrial thickness after treatment with EA in infertile women with thin endometrium. Besides, the effect of acupuncture varies with different acupoint selection. The aim of the research was to clarify the effects of EA on thin endometrium in infertile women. Therefore, the change in endometrial thickness will be used as an evaluation index. Based on our pilot study, changes in endometrial thickness of the mid-luteal phase after a 12-week treatment were shown to be 8.30 ± 2.83 mm (*n* = 10) in the electroacupuncture group (EA) and 7.10 ± 1.97 mm (*n* = 9) in the sham electroacupuncture group (SEA). Measurement data of parallel control trials (1:1) will be analyzed statistically using the following formula for sample size estimation:

$$ n=\frac{{\left({Z}_{\alpha }+{Z}_{\beta}\right)}^2\ast 2{\sigma}^2}{\delta^2} $$

With a 5% significance level (*α* = 0.05, two-sided) and 80% power (*β* = 0.2), at least 62 patients should be enrolled in each group and a total of 142 participants will be recruited to allow for a 15% dropout rate.

#### Data management and monitoring

This study will use four levels of data monitoring to improve quality control. A quality controller will check the relevant data in detail on the original CRFs and medical records regularly. The supervision’s main content is whether the initial medical history is accurate, whether the CRF form is real, and whether the correction is standardized. Supervisors will audit the accuracy of registered information, data entry, and outcomes. Any errors required that the audit be repeated from the beginning. The principal investigator will evaluate whether the recruitment and intervention procedures are conducted with rigorous adherence to the protocol. An independent data monitoring committee (DMC) consisting of three experts (an epidemiologist, an acupuncture expert, and a gynecologist) will monitor and evaluate the recruitment of subjects, the progress of research implementation, and the data’s safety, confidentiality, and integrity monthly. DMC will also regularly submit audit reports to the project management department and provide recommendations to continue the clinical trial, modify the trial protocol, or terminate the trial.

#### Statistical analysis

Statistical analyses of data will be performed using SPSS21.0 software (International Business Machines Corp., Armonk, NY) by analysts who will be blinded to the patients’ allocation. To evaluate the curative effect in this trial, an intention-to-treat analysis (ITT) will be performed. Categorical variables will be presented by frequency (percentage) and analyzed with the chi-squared test or Fisher’s exact test. Continuous variables will be presented as mean ± standard deviation (M ± SD) if they meet normal distribution. Otherwise, they will be presented as medians ± interquartile range (M ± IQR). The demographic characteristics will be compared between the groups by independent *t* tests at baseline. All longitudinal analyses for continuous variables measured at baseline and three-time periods will be performed using linear mixed modeling. Comparisons between the intervention and control groups will be performed using the chi-square test or Fisher s exact test, as appropriate, for categorical variables. To evaluate the safety of acupuncture, we will use a Fisher exact test to report the relative risk of an adverse effect. All the tests will be two-sided, and a *P* value of less than 0.05 will be considered to be statistically significant.

#### Quality control and trial monitoring

To guarantee the quality of the study, all researchers especially the acupuncturists will attend a series of training courses. The training includes how to use the randomization method, fill the case report form, manipulate interventions correctly, and other details of the trial. They will be required to master all of the details of the trial and pass a test before performing it. SOPs will be provided to practitioners to ensure identical treatment procedures. Acupuncturists who have been certified by the Ministry of Health of the People’s Republic of China and have more than 5 years of clinical experience will be recruited to perform the procedures on the participants in the study.

Other therapies that may affect the endometrial thickness and receptivity will be prohibited during treatment, such as exogenous estrogen, low-dose of aspirin, and vaginal sildenafil citrate application. Any treatment information for other diseases should be recorded in detail. To improve compliance of the participants, the principle of voluntary participation, a treatment record sheet that facilitates real-time dynamic management, traffic compensation, and pregnancy knowledge education will be considered. Participants in the SEA group may get 36 sessions of electroacupuncture free of charge after the trial.

## Discussion

### Shortcoming of other therapies

Although there are many current treatment strategies for thin endometrium, they have some unsatisfactory deficiencies and lack sufficient evidence of efficacy. Exogenous estrogen treatment is the most widely used and convenient method [10]; the risk of estrogen therapy in promoting endometrial and breast neoplasms has been clarified over the past 3 to 4 decades [40, 41]. In addition, a clinical trial revealed that estradiol supplementation starting on the human chorionic gonadotropin (HCG) day for the patients with thin endometrium did not provide any benefit on the pregnancy outcome in intracytoplasmic sperm injection (ICSI) cycles [42]. Aspirin therapy has not resulted in an improvement in the endometrial thickness, and the resistance of uterine and ovarian flow [43, 44]. Although previous data suggest that G-CSF intrauterine infusion may improve endometrial thickness, no controlled studies have demonstrated that this approach improves clinical pregnancy rates or live birth rates. Moreover, the associated potential hazards or risks need to be further considered [45, 46]. The use of stem cells appears to be a very promising option for the most refractory case, such as Asherman syndrome [47]. However, stem cell therapies are in their relative infancy [45], and more research is needed to evaluate the safety, effectiveness, and cost of this modality before it can be integrated as a medical treatment for this condition [46]. A previous trial showed that PRP efficiently improved the endometrium proliferation, implantation rate, and clinical pregnancy rate [48]; however, clinical trial data is limited, with the majority being non-randomized trials [49]. A systematic review indicates that only a few randomized controlled trials support the use of sildenafil in frozen embryo transfer (ET) for enhancing endometrial growth [50]. Other treatments, such as vitamin E and l-arginine, were all base on small sample size, thus lack sufficient evidence of efficacy [51].

### Outcome measures selection

Endometrial receptivity plays a vital role in embryo-endometrium cross talk. Endometrium exhibits a short period of receptivity, known as the “window of implantation” [2]. The IW appears in the mid-secretive phase of endometrium on the 20th to 23rd day in the menstrual cycle (namely the 7th–9th day after ovulation) [52]. Therefore, markers of endometrial receptivity are tested in the mid-luteal phase (7 days after the day of ovulation) for a natural menstrual cycle in clinical practice. The ultrasound markers for evaluation of endometrial receptivity are endometrial thickness, pattern, and blood flow impedance [5]. Endometrial thickness as an essential factor in endometrial receptivity is the most extensively used method in clinical practice [53, 54]. Previous research showed a positive correlation between endometrial thickness and implantation rates in 465 cycles of oocyte donation [55]. Endometrial blood perfusion is an important factor for endometrial growth [56]. Resistance and pulsatility index of subendometrial and uterine artery are useful tools for clinicians in predicting endometrial receptivity [57]. High blood flow impedance of uterine radial arteries is associated with poor endometrial growth in patients with thin endometrium [21]. The diastolic flow of the uterine artery in the mid-luteal phase is also associated with reproductive success [58]. Endometrial blood flow directly reflects the microenvironment of embryo implantation. Successful intrauterine implantation is positively associated with lower subendometrial resistance index (RI) and pulsatility index (PI) [59]. In the study, endometrial blood perfusion will be assessed using the PI and RI of bilateral uterine artery as well as endometrium blood flow. The endometrial patterns including type A (triple-line), type B, and type C is classified according to morphology of the endometrium [60]. An earlier study showed that the presence of a triple-line endometrium with a thickness > 6 mm was strongly correlated with conception [61]. The triple line pattern assessed on the day of HCG injection OR IUI was associated with higher clinical pregnancy rates [54]. The endometrium undergoes complex changes in the circulation of estrogen and progesterone (P), which culminate at the mid-luteal phase of the menstrual cycle when it becomes receptive [2, 62, 63]. Serum estradiol and progesterone concentrations may reflect endometrial status [64]. Estradiol stimulates both increases in the size and number of myometrial and endometrial cells as well as changes in endometrial thickness [64]. It is noteworthy that there is a minimum concentration of progesterone which is required to induce normal secretory endometrial development and receptivity [65]. This is supported by a previous study which revealed a significant decrease in clinical pregnancy and live birth rate with serum progesterone level < 10 ng/ml [66].

### The selected acupoints

In this trial, acupoints selection follows TCM theory or published articles. According to TCM theory, thin endometrium in infertile women combines with the deficiency of kidney essence, poor circulation of Qi and blood, and disharmony of thoroughfare and conception vessels. Based on the theory of acupuncture, RN3, RN4, and RN6 are located in the lower abdomen of the Ren meridian, which means dominating pregnancy, tonifying original Qi and strengthening the kidney. LR3 combines with LI4, which are called Si-guan points to regulate Qi circulation. SP6 is considered a classic acupoint for curing gynecological diseases. ST36 can facilitate the production of “qi” and “blood,” while KI3 can nourish yin and kidney essence. Currently known, acupuncture mainly improves the endometrial receptivity by increasing endometrial blood perfusion or regulating hormone receptors [27, 32, 67]. Previous study indicated that stimulation of SP6 and ST36 by electroacupuncture improved implantation rate significantly in COH rats, which may indirectly influence the secretion of estrogen and progesterone as well as facilitate the endometrial angiogenesis [25]. Another study revealed that transcutaneous electrical acupuncture point stimulation (TEAS) at RN3 (CV3), RN4 (CV4), SP6, and EX-CA1 could have beneficial effects on ultrasound markers of endometrial receptivity [67]. EA at LR3, SP6, ST28, EX-CA1, RN6, and RN4 was able to reduce uterine artery blood flow impedance in patients undergoing in vitro fertilization [68]. Warm needling at ST25, CV4, CV3, EX-CA1, and ST36 could regulate endometrial morphology and blood flow to improve endometrial receptivity, embryo transplantation rate, and pregnancy rate as well as decrease early abortion rate [69]. Warm acupuncture may promote the clinical pregnancy rate by improving endometrial blood perfusion and endometrial receptivity at the points of Qihai (CV6), Guanyuan (CV4), Zhongji (CV3), Guilai (ST29), Zigong (EX-CA1), Zusanli (ST36), and Sanyinjiao (SP6) [31]. Finally, SP10, SP8, ST29, and EX-CA1 are recommended to promote embryo implantation by consensus among experts in China [69].

This study has some limitations. Firstly, the acupuncturists cannot be blinded due to the nature of the intervention. Secondly, estimation of sample size of the proposed study was based on our previous clinical observation of a small sample size. Nevertheless, the results from this trial will provide evidence of EA as a possible therapy for thin endometrium in infertile women.

## Trial status

The recruitment of patients will start on March 1, 2020, and it is expected that by December 31, 2020, the required sample size will be reached. This is the first version, and the protocol was approved on 18 February 2020.

## Supplementary Information

**Additional file 1.**

## Data Availability

Data sharing is not applicable to this article as no datasets were analyzed during the current study. During the trial, all patient data will be accessible only to the research team.

## Abbreviations

EA: Electroacupuncture
SEA: Sham electroacupuncture
RI: Resistance index
PI: Pulsatility index
E2: Estradiol
P: Progesterone
RCT: Randomized controlled trial
IVF: In vitro fertilization
FER: Frozen embryo replacement
ICSI: Intracytoplasmic sperm injection
HCG: Human chorionic gonadotropin
ET: Embryo transfer
ER: Estrogen receptor
VEGF: Vascular endothelial growth factor
BMSCs: Bone marrow mesenchymal stem cell
CONSORT: Consolidated Standards of Reporting Trials
SPIRIT: Standardized Protocol Items: Recommendations for Interventional Trials
STRICTA: Standards for Reporting Interventions in Clinical Trials of Acupuncture
EMT: Endometrial thickness
HIV: Human immunodeficiency virus
TCM: Traditional Chinese Medicine
CRF: Case report form
DMC: Data monitoring committee
ITT: Intention-to-treat
M ± SD: Mean ± standard deviation
M ± IQR: Medians ± interquartile range
SOPs: Standard operating procedures
HPG: Hypothalamic-pituitary-gonad
LIF: Leukemia-inhibitor factor
OPN: Osteopontin
IW: Implantation window
COH: Controlled ovarian hyperstimulation
IUI: Intra-uterine insemination
TEAS: Transcutaneous electrical acupuncture point stimulation
MC: Menstrual cycle
