# Supplementary material for: Effectiveness of electroacupuncture for thin endometrium in infertile women: study protocol for a single-blind, randomized controlled trial
Source: Trials. 2021 Jan 21;22:73. doi: 10.1186/s13063-021-05029-7 (PMC7818916; doi:10.1186/s13063-021-05029-7)
Supplement: Supplementary file 1 — Additional file 1. [file 13063_2021_5029_MOESM1_ESM.docx]

**Consent Form**

**Research name**:Effectiveness of electroacupuncture for thin endometrium in infertile women:a single-blind, randomized controlled trial

**Research number**:grant number 2018YFC1704305

**Research institute**:Chengdu University of Traditional Chinese Medicine Affiliated Hospital;Chengdu University of Traditional Chinese Medicine

You will be invited to participate in a clinical study. This informed consent gives you some information to help you decide whether to participate in this clinical study or not. Please read it carefully. If you have any questions, please ask the researchers responsible for the study.

Your participation in this study is voluntary.This study has been reviewed by the ethics review committee of the research institute. If you have questions related to the subjects' rights and interests, please contact theSichuan Regional Ethics Review Committee on Traditional Chinese Medicine. Email: ethicscd@126.com.

Research purpose: This proposed randomized controlled trial (RCT), intends to evaluate the efficacy of electroacupuncture in improving endometrial receptivity of infertile women with a thin endometrium.

Research process: If you agree to participate in this study, we will number each subject and create a medical record file.You will receive 36 sessions of electroacupuncture or sham electroacupuncture over three menstrual cycles (12 weeks in total).

Risk and Privacy issue: Risks associated with this study are minimal. if you decide to participate in this study, your personal data in and during the study are confidential. You may choose not to participate in this study, or at any time inform the researcher to request withdrawal from the study. Your data will not be included in the study results, and any medical treatment and benefits will not be affected.

***If you'd like to take part in the study, You can sign this informed consent:***

Patient's name：________________________

Signature of patient：____________________

Date：

Researcher's name:____________________

Signature of researcher:____________________

Date:
